# Sirolimus Versus Mycophenolate Mofetil in Simultaneous Pancreas–Kidney Transplantation: Impact on Urinary Tract Infection Rates

**DOI:** 10.1155/joot/6985179

**Published:** 2026-04-01

**Authors:** Barbora Píšová, Šárka Chytilová, František Saudek, Peter Girman

**Affiliations:** ^1^ Department of Diabetes, Institute of Clinical and Experimental Medicine, Prague, Czech Republic, ikem.cz; ^2^ Department of Data Science, Institute of Clinical and Experimental Medicine, Prague, Czech Republic, ikem.cz

**Keywords:** immunosuppression, kidney graft survival, mycophenolate mofetil, pancreas transplantation, sirolimus, urinary tract infection

## Abstract

**Background:**

Urinary tract infections (UTIs) are common complications following simultaneous pancreas and kidney transplantation (SPK). The role of specific immunosuppressive agents in modulating UTI incidence and recurrence remains poorly understood.

**Methods:**

In this retrospective, single‐center study, we analyzed 164 SPK recipients randomized to receive either sirolimus or mycophenolate mofetil (MMF) in combination with tacrolimus. The incidence of UTIs, relapses, recurrences, and UTI‐related hospitalizations was assessed over a 10‐year follow‐up. Univariable and multivariable negative binomial regression models were used to evaluate associations with immunosuppressive regimens and clinical outcomes in both intention‐to‐treat (ITT) and per‐protocol analyses.

**Results:**

A total of 572 UTI episodes were recorded during follow‐up (0.102 per 100 recipient‐transplant days). No significant differences in overall UTI incidence or UTI‐related hospitalizations were observed between the sirolimus and MMF groups. However, in the multivariable per‐protocol analysis, the sirolimus group experienced significantly fewer UTI‐related hospitalizations (IRR 0.46; 95% CI, 0.23–0.94; *p* = 0.034) and relapses (IRR 0.54; 95% CI, 0.30–0.97; *p* = 0.039). Significant risk factors for UTIs included female sex, JJ stent placement, and pretransplant urological abnormalities. Recurrent UTIs were associated with lower 10‐year kidney graft survival (52% vs. 75%; *p* = 0.01) but had no impact on pancreas graft or patient survival.

**Conclusions:**

While overall UTI incidence did not differ between immunosuppressive regimens, sirolimus use was associated with fewer hospitalizations and relapses. These findings indicate a potential clinical benefit of sirolimus in selected high‐risk SPK recipients, highlighting the need for further prospective investigation.

**Trial Registration:** ClinicalTrials.gov identifier: NCT00140543

## 1. Introduction

Urinary tract infections (UTIs) represent the most frequent infectious complications among recipients of simultaneous pancreas and kidney transplantation (SPK), affecting nearly one‐third of patients within the first year post‐transplantation [1], and up to 63%–75% during long‐term follow‐up, as reported in single‐center studies [2, 3]. In kidney transplantation alone, the epidemiology of UTIs is more extensively documented, although reported rates vary considerably. A meta‐analysis estimated a pooled prevalence of 38% [4], with UTIs accounting for approximately 40%–50% of post‐transplant infections overall [5, 6].

It has been hypothesized that SPK recipients may experience a higher incidence of UTIs compared to diabetic patients undergoing kidney transplantation alone. However, in a study assessing infection‐related hospitalizations and episodes of bacteremia, no significant difference was observed between the two groups over a 5‐year follow‐up period [7]. Similarly, a Spanish cohort study reported a lower rate of UTIs per 100 transplant days in SPK recipients compared to kidney‐alone recipients (2.2 vs. 4.5 episodes over a 2‐year follow‐up period) [8], although that cohort also included nondiabetic patients with end‐stage renal disease.

The influence of immunosuppressive agents on UTI risk remains poorly characterized. Some studies in kidney transplant recipients have suggested that mycophenolate mofetil (MMF) may be associated with an increased UTI risk, whereas calcineurin inhibitors and mammalian target of rapamycin (mTOR) inhibitors have not shown a consistent association [9]. However, Cochrane reviews have not identified any significant difference in UTI incidence when comparing MMF to azathioprine [9] or tacrolimus to cyclosporine [10].

Sirolimus (SIRO), an mTOR inhibitor, has demonstrated efficacy as part of tacrolimus‐based immunosuppressive regimens [11, 12]. Beyond its immunosuppressive function, SIRO has also been associated with antineoplastic, antiviral, and antifungal properties [11, 13, 14]. Its antiviral activity is of particular interest in transplant recipients, with evidence of efficacy against BK virus and cytomegalovirus (CMV), both of which may rely on mTOR pathway activation for replication [15–17].

Given this background, the present study aimed to determine whether the use of SIRO, in combination with tacrolimus, reduces the incidence and recurrence of UTIs in SPK recipients compared to MMF‐based regimens.

## 2. Materials and Methods

### 2.1. Study Design

We retrospectively evaluated the incidence of UTIs in patients originally enrolled in a single‐center, open‐label, randomized trial that primarily compared patient, pancreas, and kidney graft survival between MMF and SIRO as part of tacrolimus‐based immunosuppressive regimens [12]. The frequency of UTI episodes, including relapses, recurrences, and UTI‐related hospitalizations, was assessed. In addition, this study aimed to identify risk factors associated with UTIs and their recurrences in SPK recipients, as well as to evaluate their impact on long‐term graft and patient survival.

All clinical and laboratory data were retrieved from the institutional electronic medical records. Retrospectively, all positive urine cultures were reviewed and classified as UTIs when antibiotic therapy was prescribed concurrently.

All analyses were performed using an intention‐to‐treat approach. However, given the extended observation period and the fact that some patients modified their immunosuppressive regimens for various reasons, selected outcomes were also reviewed retrospectively using a per‐protocol approach.

### 2.2. Study Population

Patients included in the present analysis were participants of a previously conducted single‐center, prospective, randomized trial comparing tacrolimus‐based immunosuppression with either SIRO or MMF in SPK. The current study represents a retrospective post hoc analysis of UTIs and related outcomes within this well‐defined cohort.

All recipients who met the inclusion criteria and consented to participate were enrolled in the study and underwent SPK at our center between January 2007 and December 2013 (*n* = 164). Eligibility criteria were previously described in detail [12]. Recipient inclusion criteria were: type 1 diabetes mellitus confirmed by history and negative or nearly negative C‐peptide level, end‐stage renal disease defined as serum creatinine > 250 μmol/L and/or creatinine clearance rate < 0.5 mL/s or regular dialysis, age > 18 years, signed informed consent.

Main exclusion criteria were: any other type of diabetes, known hypersensitivity to study drugs, any previous transplant, positive crossmatch, current malignancy or history of malignancy within the last 5 years, pregnancy or lactation, known active liver disease, severe psychiatric disorder, cardiac failure acute infection, noncompliance. All consecutive patients who were enrolled since January 2007, when the electronic documentation of UTIs and microbiological findings were implemented, were included into this retrospective analysis. In all study subjects, enteric drainage of the pancreatic graft was used. Follow‐up duration was set at 10 years post‐transplant.

Pancreas graft loss was defined as patient death, graft pancreatectomy, or return to an intensified insulin regimen. Kidney graft loss was defined as patient death, nephrectomy, or resumption of dialysis.

### 2.3. Randomization

Participants were preoperatively randomized to receive immunosuppressive treatment with either tacrolimus + SIRO or tacrolimus + MMF. Randomization was performed by the sequential opening of sealed envelopes prepared by the Department of Statistics at the Institute for Clinical and Experimental Medicine. In cases of drug intolerance or serious adverse events, the immunosuppressive regimen was modified to a better tolerated alternative.

### 2.4. Immunosuppression Protocol

In both groups, induction therapy was the same and consisted of polyclonal anti‐T‐lymphocyte globulin (ATG Fresenius/Grafalon) administered at 8 mg/kg prior to unclamping the vascular anastomoses, followed by three additional doses of 3 mg/kg daily for three consecutive days post‐transplant.

Tacrolimus was initiated at 0.1 mg/kg prior to transplantation and then continued at 0.05 mg/kg twice daily. Dose adjustments were made to maintain target trough levels of 10–15 ng/mL during the first month and 5–10 ng/mL thereafter. Methylprednisolone (250 mg) was given before transplantation, followed by three doses of 125 mg. Oral prednisone (20 mg/day) was then initiated and gradually tapered, with complete withdrawal six weeks post‐transplant.

Antimicrobial prophylaxis was identical in both groups and included: piperacillin/tazobactam 4.5 g three times daily for 4 days; fluconazole 100 mg daily for 1 week; valganciclovir 450 mg daily for 6 months; and trimethoprim/sulfamethoxazole 960 mg twice weekly for 9 months.

• **MMF group:** MMF was administered at a fixed dose of 2 g per day. In case of leucopenia or gastrointestinal intolerance, the dose was temporarily or permanently decreased.
• **SIRO group:** SIRO was initiated at 5 mg pretransplant, with subsequent trough levels maintained between 5 and 10 ng/mL.

### 2.5. Clinical Evaluations

Patients underwent scheduled clinical evaluations every 3 months throughout the study period. These included assessment of medical history, physical examination, graft function, complete blood count, urinalysis including microbiology, and blood levels of immunosuppressive agents (tacrolimus and SIRO). Study participants were instructed to contact the transplant center in the event of any intercurrent medical issues.

UTI was defined in accordance with the CDC criteria [18] as the presence of ≥ 10^5^ colony‐forming units (CFU)/mL in a urine specimen accompanied by clinical symptoms. This was a retrospective study. Hospitalizations were identified based on documented admission records and were classified as UTI‐related only if the UTI was the principal reason for admission.

A relapse was defined as a new episode of UTI caused by the same pathogen occurring within 3 months following completion of antibiotic therapy. A recurrence was defined as either a UTI caused by a different pathogen regardless of timing or by the same pathogen more than 3 months after the initial episode.

Hospitalization for UTI was indicated in cases of suspected acute pyelonephritis, febrile illness requiring intravenous antibiotic therapy or antibiotics available exclusively in parenteral formulations, or in the presence of clinical instability of the patient.

Urinary catheter management followed a standardized institutional protocol and did not differ between treatment arms.

### 2.6. Ethics Statement

The study was approved by the Ethics Committee of the Institute for Clinical and Experimental Medicine and Thomayer Teaching Hospital in Prague, Czech Republic. All relevant clinical and microbiological data were retrieved from institutional electronic databases and processed anonymously in accordance with applicable data protection regulations. The study was conducted in compliance with the principles of the Declaration of Helsinki.

### 2.7. Statistics Analysis

All analyses were performed on both an intention‐to‐treat and a per‐protocol basis using *R* software (version 4.3.2) and Statistica (version 12; TIBCO Software, Inc., Palo Alto, CA). Continuous variables were reported as arithmetic means with standard deviations and compared using the two‐sided non‐parametric Wilcoxon rank‐sum test (reported *p*‐values are unadjusted for multiple comparisons). Categorical variables were expressed as absolute frequencies and percentages and compared using Fisher’s exact test (*p* values also unadjusted).

To evaluate the effect of immunosuppressive agents and other potential risk factors on various UTI‐related outcomes, a multivariable negative binomial regression model with overdispersion was employed, using the glmmTMB *R* package (version 1.1.9). Results are reported as incidence rate ratios (IRRs) with associated *p* values, calculated using the gtsummary *R* package (version 1.7.2). The MMF group served as the reference.

The multivariable models included the following covariates: sex, presence of a JJ stent, history of UTI prior to transplantation, pretransplant urological complications, duration of diabetes, and dialysis dependency at the time of listing.

Survival analysis was conducted using all available follow‐up data, including observations beyond the 10‐year time point. However, Kaplan–Meier survival curves are presented only for the first 10 years. The log‐rank test for differences between immunosuppressive regimens was performed using the complete dataset.

### 2.8. Endpoint

The primary endpoint was the difference in overall UTI incidence between the SIRO and MMF treatment groups. Secondary endpoints included the identification of independent risk factors for UTI, differences in the rates of UTI relapses and recurrences, and patient and graft survival outcomes.

## 3. Results

### 3.1. Demographic Data

Baseline characteristics of the study population are summarized in Table 1. A total of 164 patients were included, with 80 patients in the SIRO group and 84 in the MMF group in the ITT analysis. In both groups, male recipients predominated, with females representing 40.0% in the SIRO group and 42.8% in the MMF group (*p* = 0.75). During follow‐up, a total of 20 patients changed their immunosuppressive regimen, resulting in 76 patients remaining in the SIRO group and 88 in the MMF arm in the per‐protocol analysis (PPA).

**TABLE 1:** Baseline demographic and clinical characteristics of the study population. Data are presented as mean ± standard deviation (SD), or as counts and percentages, as appropriate.

Demographic data	MMF (*n* = 84)	Sirolimus (*n* = 80)	*p*‐value
Time of follow‐up (years ± SD)	9.37 ± 1.92	9.43 ± 1.81	0.10
Female sex (%)	43%	40%	0.75
Recipient age (years ± SD)	43.23 ± 9.49	42.99 ± 9.49	0.87
Time on waiting list (days ± SD)	586.69 ± 393.29	586.58 ± 389.99	0.98
Diabetes duration (years ± SD)	26.67 ± 7.86	26.81 ± 8.19	0.93
Recurrent UTI in history (%)	9.5%	7.5%	0.78
Urological complication prior to transplant (%)	15.5%	17.5%	0.83
JJ stent (%)	20.24%	27.50%	0.36
Pretransplant without dialysis (%)	50.0%	32.5%	0.03
Hemodialysis (%)	42%	54%	0.16
Peritoneal dialysis (%)	8%	14%	0.32
Donor age (years ± SD)	28.39 ± 8.51	29.52 ± 8.88	0.52
Donor BMI (± SD)	23.61 ± 2.94	23.39 ± 3.16	0.49
CMV D−/R− (*n*)	5	6	0.77
CMV D−/R + (*n*)	15	14	1.00
CMV D+/R + (*n*)	41	35	0.54
CMV D+/R− (*n*)	23	25	0.61
Donor serum creatinine (μmol/L ± SD)	85.50 ± 35.40	77.67 ± 24.77	0.22
MHC mismatches (± SD)	4.40 ± 0.97	4.31 ± 1.04	0.83
0–2 HLA mismatches (*n*)	2	5	0.27
3–4 HLA mismatches (*n*)	43	37	0.54
5–6 HLA mismatches (*n*)	39	38	1.00
PRA < 20% (%)	93%	95%	0.75
PRA 21%–80% (%)	7%	5%	0.75
Pancreas donor risk index (± SD)	1.03 ± 0.31	1.07 ± 0.31	0.31
Cold ischemia time—pancreas (*h* ± SD)	10.28 ± 2.62	10.10 ± 2.67	0.55
Cold ischemia time—kidney (*h* ± SD)	12.39 ± 2.86	11.99 ± 2.66	0.32

*Note:* *p*‐values correspond to the results of the Wilcoxon rank‐sum test. In all study subjects, enteric exocrine drainage of the transplanted pancreas was used.

The mean duration of follow‐up was 9.26 ± 1.81 years in the SIRO group and 9.37 ± 1.92 years in the MMF group (*p* = 0.11). No significant differences were observed between the groups in terms of recipient or donor age, time on the waiting list, duration of diabetes, number of HLA mismatches, donor CMV serostatus, panel of reactive antibody (PRA) levels, pancreas donor risk index (PDRI), or mean cold ischemia times for kidney and pancreas grafts.

A significantly higher proportion of patients in the MMF group were pre‐emptively transplanted without prior dialysis (*n* = 42; 50.0%) compared to the SIRO group (*n* = 26; 32.5%; *p* = 0.027) (see Table 1).

### 3.2. UTI

Over the course of the study, a total of 572 UTI episodes were recorded. The overall incidence rate was 0.102 episodes per 100 recipient‐transplant days, based on a cumulative follow‐up of 1528.2 recipient‐years.

In the MMF group, 290 UTI episodes were documented (mean 3.45; SD 4.88; median 2 [IQR 0–4]) and 282 episodes occurred in the SIRO group (mean 3.53; SD 5.31; median 2 [IQR 0–4]).

Of these, 159 episodes required hospitalization, with 103 hospitalizations in the MMF group (mean 1.24; SD 3.07; median 0 [IQR 0–1]) and 56 in the SIRO group (mean 0.70; SD 1.38; median 1 [IQR 0–1]).

During the 10‐year follow‐up period, the overall mean numbers of hospitalizations for any reason were 8.62 (SD 10.18) and 7.35 (SD 5.75) in the MMF and SIRO groups, respectively (*p* = 0.805).

No significant difference in overall UTI incidence was observed between groups (IRR 1.02; 95% CI 0.66–1.58; *p* > 0.9), nor in the rate of UTI‐related hospitalizations (IRR 0.56; 95% CI 0.28–1.14; *p* = 0.11) (Table 2). Nonetheless, a downward trend in hospitalization rates was noted in the SIRO group.

**TABLE 2:** Urinary tract infections in the MMF and sirolimus groups.

Outcome	MMF (*n* = 84) Mean ± SD	Sirolimus (*n* = 80) Mean ± SD	Univariable IRR (95% CI)	*p*‐value	Multivariable IRR (95% CI)	*p*‐value
Episodes of UTI	3.45 ± 4.88	3.53 ± 5.31	1.02 (0.66–1.58)	> 0.9	0.91 (0.63–1.31)	0.6
Hospitalizations for UTI	1.24 ± 3.07	0.7 ± 1.38	0.56 (0.28–1.14)	0.11	0.50 (0.25–1.01)	0.05
UTI relapse	1.46 ± 2.35	1.11 ± 2.44	0.76 (0.41–1.42)	0.4	0.58 (0.32–1.06)	0.07
Recurrence of UTI	0.96 ± 1.67	0.97 ± 1.56	1.01 (0.59–1.72)	> 0.9	0.97 (0.62–1.52)	0.9
UTIs in patients with pre‐Tx recurrent UTI	8.00 ± 8.05	8.67 ± 7.66	1.08 (0.38–3.19)	0.9	Not done	Not done
Hospitalizations in pre‐Tx recurrent UTI	3.75 ± 7.50	2.33 ± 3.01	0.62 (0.07–6.53)	0.7	Not done	Not done

*Note:* Results of univariable and multivariable analysis in the intention‐to‐treat analysis. IRR: Incidence rate ratio. Not done: not performed due to low number of observations.

Among patients with a history of recurrent UTIs prior to transplantation, there was no significant difference in the number of post‐transplant UTIs between the two groups (IRR 1.08; 95% CI 0.38–3.19; *p* = 0.90) nor in hospitalization rates (IRR 0.62; 95% CI 0.07–6.53; *p* = 0.7).

**FIGURE 1:**
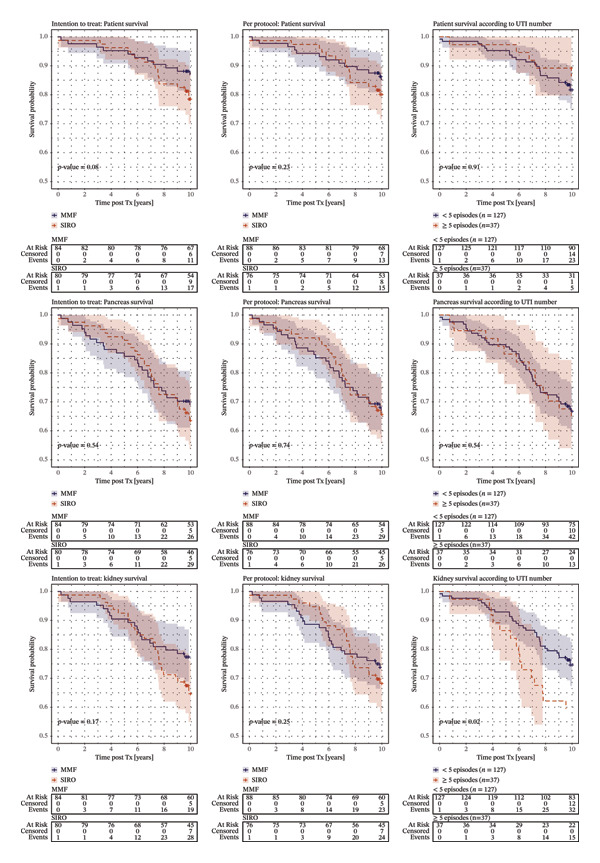
Patient, kidney graft, and pancreas graft survival with regard to immunosuppressive therapy, intention‐to‐treat and per‐protocol analysis, and the number of urinary tract infection episodes.

Notably, significantly fewer patients in the SIRO group experienced more than two UTI relapses compared to the MMF group (17.5% vs. 30.0%; *p* = 0.05). Figure 1, panel I demonstrates significantly worse kidney graft survival rate in subjects with five or more relapses. Of note, among this group in the PPA, there were significantly more subjects treated with MMF than with SIRO (26 versus 16, *p* = 0.01).

### 3.3. Risk Factors

Statistically significant risk factors for developing UTI after SPK included female sex (IRR 5.45; 95% CI 3.58–8.30; *p* < 0.001), intraoperative insertion of a JJ stent (IRR 1.86; 95% CI 1.24–2.79; *p* = 0.003), and the presence of urological abnormalities identified during pretransplant evaluation requiring urological intervention (IRR 1.77; 95% CI 1.07–2.91; *p* = 0.026).

By contrast, a history of recurrent UTIs prior to transplantation, recipient age, duration of diabetes, and pretransplant dialysis status were not associated with a significantly increased risk of post‐transplant UTI (see Table 3).

**TABLE 3:** Risk factors for urinary tract infections.

	**IRR**	**95% CI**	**p**

Female	5.26	3.58–8.30	0.001
Age	1.02	1.00–1.04	0.088
JJ stent	1.86	1.24–2.79	0.003
Urological complication prior Tx	1.77	1.07–2.91	0.026
History of recurrent UTI prior TX	1.42	0.83–2.40	0.2
Duration of DM prior Tx	0.98	0.96–1.01	0.2
Dialysis prior Tx	1.08	0.76–1.55	0.7

### 3.4. Graft and Patient Survival Rates and Rejections

No significant differences were observed in 10‐year patient, pancreas, or kidney graft survival between the MMF and SIRO treatment groups. Ten‐year patient survival was identical (82%; *p* = 0.6) among patients who experienced more than five episodes of UTI during the follow‐up period (INF + (patients with ≥ 5 UTIs)) and those with fewer episodes (INF − (patients with < 5 UTIs)).

While the cumulative number of UTIs had no significant impact on pancreas graft survival (66% vs. 65%; *p* = 0.3), it was significantly associated with reduced kidney graft survival (75% in INF − (patients with < 5 UTIs) vs. 52% in INF + (patients with ≥ 5 UTIs); *p* = 0.01). See Figure 1.

Biopsy‐proven pancreas rejection was more frequent in the MMF group than in the SIRO group (20.2% vs. 8.6%; *p* = 0.037). However, there were no differences between the rates of all rejections (33% and 27.5%; *p* = 0.2) or kidney graft rejections (15.5% and 18.6%; *p* = 0.73) in the MMF and SIRO groups, respectively. In the whole patient group (treated with MMF or SIRO), the presence of any kind of rejection did not significantly correlate with the occurrence or UTI, relapse or reinfection (Spearman correlation).

Among patients who developed at least one UTI, 16 episodes of treated acute kidney and/or pancreas rejection were recorded in the MMF group, compared with 9 episodes in the SIRO group.

### 3.5. PPA and Immunosuppression Changes

A total of 20 patients (12.2%) changed their immunosuppressive regimen during the study period, with the majority (*n* = 17/20; 85%) making the change within the first year post‐transplant. Twelve patients transitioned from SIRO to MMF, most commonly due to adverse effects such as impaired wound healing (*n* = 5) and leukopenia (*n* = 2). Eight patients switched from MMF to SIRO, with malignancy being the leading indication (*n* = 2), while the remaining switches were due to various other clinical considerations.

For the PPA, 88 patients from the MMF group and 76 from the SIRO group were included. The incidence rates of UTI episodes were not significantly different between groups (IRR 0.68; 95% CI 0.44–1.06; *p* = 0.10). However, patients treated with SIRO experienced significantly fewer hospitalizations (IRR 0.47; 95% CI 0.23–0.94; *p* = 0.03) and showed a trend toward fewer relapses (IRR 0.57; 95% CI 0.31–1.06; *p* = 0.08). In contrast, UTI recurrences were not significantly different (IRR 0.65; 95% CI 0.38–1.10; *p* = 0.11).

In multivariate analysis, these findings were confirmed, showing a significantly lower risk of both hospitalizations due to UTI (IRR 0.46; 95% CI 0.23–0.94; *p* = 0.03) and UTI relapse (IRR 0.54; 95% CI 0.30–0.97; *p* = 0.04) in the SIRO group (see Table 4).

**TABLE 4:** Per‐protocol analysis of UTI‐related outcomes.

Outcome	MMF (*n* = 88) Mean ± SD	Sirolimus (*n* = 76) Mean ± SD	Univariable IRR (95% CI)	*p*‐value	Multivariable IRR (95% CI)	*p*‐value
Episodes of UTI	04.08 ± 5.44	2.79 ± 4.5	0.68 (0.44–1.06)	0.09	0.73 (0.51–1.06)	0.10
Hospitalizations for UTI	1.30 ± 3.02	0.61 ± 1.32	0.47 (0.23–0.94)	0.03	0.46 (0.23–0.94)	0.03
UTI relapse	1.61 ± 2.65	0.92 ± 2.00	0.57 (0.31–1.06)	0.08	0.54 (0.30–0.97)	0.04
Recurrence of UTI	1.16 ± 1.87	0.75 ± 1.21	0.65 (0.38–1.10)	0.11	0.75 (0.47–1.20)	0.2

Tacrolimus and SIRO trough plasma levels were measured every 3 months throughout the whole study period. Median tacrolimus levels in the MMF group were slightly higher than in the SIRO group (8.28 ng/mL; interquartile range 7.09–8.99) versus 7.65 ng/mL; interquartile range 7.09–8,46); *p* = 0.002); Wilcoxon test *p* = 0.002. Median SIRO levels were 5.94 ng/mL in the SIRO group.

### 3.6. Viral Infections

In the present cohort, no statistically significant difference was observed in the incidence of CMV or BK polyomavirus@@ (BKV) infection between the MMF and SIRO treatment groups. BKV infection was assessed on an annual basis and defined as serum viremia ≥ 10^4^ copies/mL. BKV viremia was detected in 4 patients in the MMF group and in 10 patients in the SIRO group (*p* = 0.13). CMV testing was performed only in cases of clinical suspicion and was considered positive if the viral load exceeded 500 copies/mL or if CMV infection was confirmed by histological examination, regardless of viral load. CMV infection was identified in 11 patients treated with MMF and in 3 patients treated with SIRO (*p* = 0.06).

Coincidence of viral infection and UTI was rare. Only one patient in the MMF group experienced a UTI concurrent with CMV infection, and only one patient in the SIRO group experienced a UTI concurrent with BKV infection.

## 4. Discussion

In our study population, 68.3% of patients experienced at least one UTI during the 10‐year follow‐up period, which is consistent with previously published single‐center studies reporting long‐term UTI rates of 64%–75% among SPK recipients [2, 3]. The incidence rate of UTIs in our cohort—0.102 per 100 transplant days (572 UTI per 1582 patient years) —was lower than the 2.2 episodes per 100 transplant days reported in a Spanish registry with a shorter 3‐year follow‐up and with more than 50% subjects with bladder drainage of the pancreas [8]. This difference may reflect the well‐documented tendency for UTI incidence to peak during the first post‐transplant year [19].

In our primary analysis, no statistically significant differences were observed between the SIRO and MMF groups in the overall incidence of UTIs or UTI‐related hospitalizations. However, there was trend toward lower number of hospitalizations in the SIRO arm. Female sex and a history of infection prior to transplantation were also associated with higher hospitalization rates.

Given the long duration of follow‐up and the rate of immunosuppressive regimen changes, we also performed a PPA that accounted for post‐transplant treatment modifications, recipient sex, and history of infection prior to transplantation. This analysis may have unmasked the effect of treatment and confirmed the reduction in hospitalizations and relapses among patients maintained on SIRO, with significantly fewer individuals experiencing five or more UTI episodes during follow‐up. The overall incidence of UTIs remained comparable between groups in both ITT analysis and PPA.

The mechanistic basis for the observed reduction in UTI‐related hospitalizations and relapses among SIRO ‐treated patients remains incompletely understood. One possible explanation relates to differences in pharmacokinetic management between the two regimens. SIRO dosing is routinely individualized using therapeutic drug monitoring, whereas MMF is generally administered at a fixed dose without systematic exposure‐guided adjustment. This may result in greater interindividual variability in the net state of immunosuppression in MMF‐treated patients.

While median tacrolimus levels were statistically significantly higher in the MMF group, the absolute difference was small and unlikely to be clinically relevant (8.28 vs 7.65 ng/mL). In our previously published study, which included a larger cohort of patients, we observed a higher incidence of rejection episodes in those treated with MMF during the 5‐year follow‐up (34% versus 21%; *p* = 0.029). In the present analysis, however, we noted only a more frequent occurrence of isolated pancreas rejections, whereas the overall number of rejection episodes did not differ significantly between the groups. We therefore believe that the more frequent occurrence of severe UTIs in the MMF‐treated group was unlikely to have been caused by escalation of immunosuppressive therapy during rejection episodes.

Importantly, even when the analysis was restricted to patients with UTIs, treated rejection episodes were numerically more frequent in the MMF group, arguing against the possibility that more intensive immunosuppressive treatment in the SIRO group contributed to the observed differences in UTI‐related outcomes.

Beyond this pharmacological aspect, additional biologically plausible mechanisms should be considered. mTOR inhibitors have consistently been associated with a reduced incidence and severity of viral infections in solid organ transplant recipients, particularly CMV and BK polyomavirus [14–17]. Both viruses rely, at least in part, on host cell mTOR signaling for replication, and inhibition of this pathway has been shown to limit viral replication and clinical disease. Subclinical or overt viral reactivation may predispose to bacterial UTIs through urothelial injury, local immune dysregulation, and the need for intensified immunosuppression or antiviral therapy. A lower burden of CMV or BK virus replication in patients treated with SIRO could therefore indirectly contribute to a reduced risk of clinically severe or recurrent UTIs.

In addition, although SIRO has no known direct antibacterial activity, mTOR inhibition may influence innate immune responses, inflammatory signaling, and epithelial cell turnover, potentially affecting the integrity of the urothelial barrier and host susceptibility to infection. Finally, a lower frequency of treatment escalation due to rejection episodes, as observed in some SIRO ‐based regimens, may limit fluctuations in immunosuppressive intensity and thereby reduce the risk of severe infectious complications.

In our cohort, however, we did not observe a statistically significant difference in the incidence of CMV or BKV infection between the treatment groups. Moreover, concurrent occurrence of viral infection and UTI was rare, suggesting that active CMV or BKV replication was unlikely to be a major direct driver of UTI episodes in this population. These findings indicate that, while reduced viral replication under mTOR inhibition remains a biologically plausible mechanism in other settings, it does not appear to fully explain the observed differences in UTI‐related hospitalizations and relapses in our study. Other factors, including differences in pharmacokinetic management, host immune response, or local urothelial susceptibility, may therefore play a more prominent role.

These hypotheses remain speculative and cannot be definitively addressed by the present study; however, they provide a biologically plausible framework for the observed differences in UTI‐related outcomes and warrant further investigation in prospective studies incorporating detailed virological and immunological monitoring.

Risk factors for UTI after SPK are multifactorial, involving patient‐related factors, immunosuppressive agents, surgical technique, and anatomical considerations [20]. Previous studies have implicated female sex, bladder drainage of the exocrine pancreas, longer diabetes duration, and urological pathology as significant predictors of recurrent UTI [2, 9, 18, 20–22]. In our study, only female sex, intraoperative JJ stent placement, and pretransplant urological abnormalities requiring intervention were independently associated with increased UTI risk. Notably, diabetes duration and pretransplant history of recurrent UTIs did not emerge as statistically significant risk factors in our cohort. A clinically relevant question is whether JJ stent placement merely reflects a subgroup of patients with more complex urological anatomy rather than representing an independent risk factor. In our cohort, pretransplant urological abnormalities and intraoperative JJ stent insertion were recorded separately and did not fully overlap. Urological abnormalities typically included conditions such as neurogenic bladder dysfunction, vesicoureteral reflux, urethral strictures, or bladder outlet obstruction requiring intervention before transplantation, whereas JJ stents were placed intraoperatively according to the surgeon’s assessment of ureteral anastomosis protection.

These findings suggest that JJ stent placement likely represents a procedural risk factor related to temporary foreign body colonization rather than solely a marker of pre‐existing urological pathology, although some degree of interaction between the two cannot be excluded.

UTIs remain among the most common infectious complications following SPK [1, 18, 21] and have been associated with adverse effects on graft function and long‐term outcomes in kidney transplant recipients [21–24]. Our study supports these findings: patients who experienced ≥ 5 UTIs within 10 years post‐transplant had significantly lower kidney graft survival. In contrast, no such effect was observed on pancreas graft survival, in accordance with earlier SPK studies [21]. Ten‐year patient survival was unaffected by UTI burden. It is important to note that we did not distinguish between lower UTIs (e.g., cystitis) and upper tract infections (e.g., pyelonephritis), which have different clinical implications and may affect graft function differently [24].

Although SIRO has gained broader clinical use due to its immunosuppressive efficacy and non‐nephrotoxic profile, its benefits are often offset by a high rate of adverse effects, including impaired wound healing, myelosuppression, and hyperlipidemia [11, 13, 14]. In previous trials, SIRO discontinuation rates have ranged from 20% to 30% [14]. In our cohort, only 15% of patients discontinued SIRO during the 10‐year follow‐up, most commonly due to impaired wound healing. Patient and graft survival outcomes were comparable between MMF and SIRO recipients, consistent with our previously reported findings [12].

## 5. Limitations

Several limitations of this study should be acknowledged. First and foremost, the retrospective design limits causal inference. We were unable to differentiate between lower UTIs (e.g., cystitis) and graft pyelonephritis, which may have distinct clinical courses and prognostic implications. Additionally, our reported incidence of UTI may underestimate the true burden, as some episodes treated outside our center may have gone undocumented—despite recommendations for patients to report all post‐transplant complications to our clinic.

UTI episodes were identified based on the combination of antibiotic prescription and a positive urine culture, which may not fully capture asymptomatic bacteriuria or infections treated empirically. Finally, treatment adherence and the use of prophylactic antibiotics were not systematically assessed.

## 6. Conclusion

In conclusion, patients undergoing SPK are at a high risk of developing UTIs in the post‐transplant period. Although no significant difference was observed in the overall incidence of UTI or UTI‐related hospitalizations between patients treated with MMF or SIRO, those in the SIRO group experienced significantly fewer relapses and recurrences—particularly in multivariate analysis and PPA. These findings indicate that SIRO may offer clinical benefit for SPK recipients with recurrent UTIs. However, this hypothesis warrants evaluation in prospective, randomized trials.

Furthermore, our study confirms that recurrent UTIs negatively impact long‐term kidney graft survival, although they do not appear to influence patient survival or pancreas graft outcomes.

## Funding

This study was supported by Ministry of Health, Czech Republic—conceptual development of research organization (“Institute for Clinical and Experimental Medicine—IKEM, IN 00023001”) and by the project National Institute for Research of Metabolic and Cardiovascular Diseases (Programme EXCELES, ID Project No. LX22NPO5104)—Funded by the European Union – Next Generation EU.

## Disclosure

The results of this manuscript were partially presented at the 13^th^ EPITA symposium, Igls, Austria, January 2024 (see Abstract at: https://esot.org/wp-content/uploads/2024/01/EPITA_2024_Abstract_book_270124.pdf).

## Conflicts of Interest

The authors declare no conflicts of interest.

## Data Availability

The data that support the findings of this study are available upon request from the corresponding author. The data are not publicly available due to privacy or ethical restrictions.
